# Palbociclib impairs the proliferative capacity of activated T cells while retaining their cytotoxic efficacy

**DOI:** 10.3389/fphar.2023.970457

**Published:** 2023-02-03

**Authors:** Claudia Arndt, Antje Tunger, Rebekka Wehner, Rebecca Rothe, Eleni Kourtellari, Stephanie Luttosch, Katharina Hannemann, Stefanie Koristka, Liliana R. Loureiro, Anja Feldmann, Torsten Tonn, Theresa Link, Jan Dominik Kuhlmann, Pauline Wimberger, Michael Philipp Bachmann, Marc Schmitz

**Affiliations:** ^1^ Department of Radioimmunology, Institute of Radiopharmaceutical Cancer Research, Helmholtz-Zentrum Dresden-Rossendorf, Dresden, Germany; ^2^ Mildred Scheel Early Career Center, Faculty of Medicine Carl Gustav Carus, TU Dresden, Dresden, Germany; ^3^ National Center for Tumor Diseases (NCT), University Hospital Carl Gustav Carus, TU Dresden, Dresden, Germany; ^4^ Institute of Immunology, Faculty of Medicine Carl Gustav Carus, TU Dresden, Dresden, Germany; ^5^ German Cancer Consortium (DKTK), Partner Site Dresden, German Cancer Research Center (DKFZ), Heidelberg, Germany; ^6^ German Red Cross Blood Donation Service North-East, Institute for Transfusion Medicine, Dresden, Germany; ^7^ Experimental Transfusion Medicine, Faculty of Medicine Carl Gustav Carus, TU Dresden, Dresden, Germany; ^8^ Department of Gynecology and Obstetrics, University Hospital Carl Gustav Carus, TU Dresden, Dresden, Germany; ^9^ Tumor Immunology, University Cancer Center (UCC), University Hospital Carl Gustav Carus, TU Dresden, Dresden, Germany

**Keywords:** cancer immunotherapy, CDK4/6, palbociclib, fulvestrant, bispecific antibody, CAR T cell, adoptive T cell therapy

## Abstract

The cyclin-dependent kinase 4 and 6 (CDK4/6) inhibitor palbociclib is an emerging cancer therapeutic that just recently gained Food and Drug Administration approval for treatment of estrogen receptor (ER)-positive, human epidermal growth factor receptor (Her)2-negative breast cancer in combination with the ER degrader fulvestrant. However, CDK4/6 inhibitors are not cancer-specific and may affect also other proliferating cells. Given the importance of T cells in antitumor defense, we studied the influence of palbociclib/fulvestrant on human CD3+ T cells and novel emerging T cell-based cancer immunotherapies. Palbociclib considerably inhibited the proliferation of activated T cells by mediating G0/G1 cell cycle arrest. However, after stopping the drug supply this suppression was fully reversible. In light of combination approaches, we further investigated the effect of palbociclib/fulvestrant on T cell-based immunotherapies by using a CD3-PSCA bispecific antibody or universal chimeric antigen receptor (UniCAR) T cells. Thereby, we observed that palbociclib clearly impaired T cell expansion. This effect resulted in a lower total concentration of interferon-γ and tumor necrosis factor, while palbociclib did not inhibit the average cytokine release per cell. In addition, the cytotoxic potential of the redirected T cells was unaffected by palbociclib and fulvestrant. Overall, these novel findings may have implications for the design of treatment modalities combining CDK4/6 inhibition and T cell-based cancer immunotherapeutic strategies.

## 1 Introduction

Approximately 80% of breast cancers are hormone receptor-positive (HR+) and therefore represent the largest subtype of this malignancy. Endocrine therapies targeting the estrogen receptor (ER) using aromatase inhibitors, such as letrozole, preventing ER signaling (Finn et al., 2015; Finn et al., 2016b; Goetz et al., 2017), selective ER degraders, like fulvestrant (Turner et al., 2015; Sledge et al., 2017; Turner et al., 2018), or selective ER modulators as tamoxifen (Tripathy et al., 2018b) substantially reduced tumor recurrence and improved overall survival (OS) (Abe et al., 2005). However, a significant proportion of patients suffers from relapse following single-agent treatment (Abe et al., 2005; Baselga et al., 2012). To overcome resistance to endocrine therapy new treatment options were developed, such as cyclin-dependent kinase 4 and 6 (CDK4/6) inhibitors, which significantly improved clinical outcomes for these patients (Finn et al., 2015; Turner et al., 2018). CDK4/6 are fundamental drivers of the cell cycle by regulating initiation and progression through the G1 phase and are therefore also key players in various malignancies (Yu et al., 2006; Choi et al., 2012). Common dysregulations of the CDK4/6-retinoblastoma protein (Rb) axis, like copy-number variation or overexpression as well as loss of negative regulators of the pathway, can lead to cancer formation (Sherr et al., 2016). Accordingly, CDKs have long been attractive targets for pharmacologic inhibition in tumor therapy (Adams et al., 2015; Sherr et al., 2016).

The cytostatic potential of single-agent CDK4/6 inhibitors has been shown *in vitro*, causing downregulation of transcription factor E2F target genes, loss of proliferation markers and cell cycle arrest in G1 (Fry et al., 2004). In particular, HR+ breast cancer is susceptible to CDK4/6 inhibitor therapy (Finn et al., 2009; O’Leary et al., 2016). Given the fact that activation of the cyclin D-CDK4/6 complex depends on mitogenic stimuli, synergistic combinations of CDK4/6 inhibitors with signal transduction inhibitors have been developed. In particular, the three orally available CDK4/6 inhibitors palbociclib (PD-0332991; Ibrance; Pfizer), ribociclib (LEE011; Kisqali; Novartis) and abemaciclib (LY2835219; Verzenio; Lilly) received approval by the Food and Drug Administration (FDA) for treatment of patients with ER+, human epidermal growth factor receptor 2-negative (HER2-) advanced or metastatic breast cancer in combination with an aromatase inhibitor or fulvestrant (Finn et al., 2016a; Cristofanilli et al., 2016; Hortobagyi et al., 2016). Various clinical trials within the framework of the PALOMA, MONALEESA, and MONARCH study families form the basis for the FDA approvals, showing improved progression-free survival (PFS) and OS for treatment with CDK4/6 inhibitors and endocrine therapy in breast cancer patients (Finn et al., 2016b; Cristofanilli et al., 2016; Tripathy et al., 2018a; Turner et al., 2018; Im et al., 2019; Johnston et al., 2020; Slamon et al., 2020; Sledge et al., 2020). Beyond ER+ breast cancer, promising activity of CDK4/6 inhibitors in mantle cell lymphoma (MCL), liposarcoma, melanoma, non-small cell lung cancer (NSCLC), glioblastoma, neuroblastoma and malignant rhabdoid tumors has been shown (Leonard et al., 2012; Dickson et al., 2016; Patnaik et al., 2016; Geoerger et al., 2017). Further studies are currently underway, e.g. the evaluation of ribociclib for treatment of prostate cancer (Scheinberg et al., 2020).

However, resistance to therapy frequently occurs in treated patients (Finn et al., 2016a; Finn et al., 2016b; Cristofanilli et al., 2016; Hortobagyi et al., 2016). For this reason, new therapeutic strategies are required to overcome the resistance to CDK4/6 inhibition. The combination with other strategies, such as immunotherapeutic approaches, may represent an interesting treatment modality. There is increasing evidence that CDKs not only regulate cell cycle progression in tumor cells, but also development, differentiation and activation of immune cells (Wells and Morawski, 2014; Ameratunga et al., 2019; Laphanuwat and Jirawatnotai, 2019). T cells play a major role in antitumor immune defense. Based on their antitumoral properties, such as production of proinflammatory cytokines and cytotoxic activity, T cells emerged as a promising tool for cancer immunotherapy. An attractive approach is the genetic modification of autologous T cells with chimeric antigen receptors (CARs) targeting tumor-associated antigens (TAAs). By this, T cells can be redirected against tumor cells in a major histocompatibility complex (MHC)-independent manner (Sadelain et al., 2013). Currently, there are several clinical trials of CAR T cells targeting HER-2 (NCT01935843, NCT01022138), cMet (NCT03060356) or mesothelin (NCT02580747) in breast cancer patients. In this study, we used the switchable UniCAR system (Bachmann, 2019), that is also currently under clinical investigation in a phase I clinical trial (NCT04230265). As an adaptor CAR system, UniCAR T cells recognize a small epitope not present on the cell surface. Thus, they are *per se* inactive and have to be combined with a tumor-reactive target module (TM) to induce tumor lysis, thereby separating the signaling and tumor-targeting function of CARs (Bachmann, 2019; Arndt et al., 2020a). Here, we are utilizing a well-established prostate stem cell antigen (PSCA)-specific TM to redirect UniCAR T cells against prostate cancer cells (Arndt et al., 2014b; 2014a). Alternative strategies to redirect T cells towards tumor cells are bispecific antibodies (bsAbs) that simultaneously target CD3 and a TAA. Due to the bsAb-mediated cross-linkage, T cells can be efficiently engaged for tumor cell killing independent of their TCR-specificity and costimulatory signals (Wolf et al., 2005; Offner et al., 2006). In a phase II study (NCT04224272), the combination of the HER-2-targeting bsAb ZW25 and palbociclib plus fulvestrant for HER2+/HR+ advanced breast cancer is under investigation. Carcinoembryonic antigen (NCT01730612) and PSCA (NCT03927573) are further antigens for targeting breast cancer cells with bsAbs. Here, we used the CD3-PSCA bsAb, which triggers an efficient T cell-mediated killing of PSCA+ tumor cells (Feldmann et al., 2012; Arndt et al., 2014b).

Based on these findings, the aim of the present study was to examine the impact of palbociclib and fulvestrant alone or in combination on CD3+ T cells and novel emerging T cell-based cancer immunotherapies. In this context, we explored the impact of these two therapeutic agents on proliferation, cytokine production and cytotoxic potential of PSCA-specific UniCAR T cells (Feldmann et al., 2017) and T cells redirected *via* CD3-PSCA bsAb (Feldmann et al., 2012).

## 2 Materials and methods

### 2.1 Cell lines

All cell lines were maintained at 37°C in a humidified atmosphere with 5% CO_2_. Recombinant antibody producing 3T3 cell lines were cultured in DMEM complete media (Feldmann et al., 2011). The prostate cancer cell lines PC3-PSCA/PSMA Luc+ and LNCaP-PSCA Luc+ were generated and cultured as previously described (Feldmann et al., 2017).

### 2.2 Production and purification of recombinant antibody constructs

Construction and cloning of PSCA TM and CD3-PSCA bsAb have been published elsewhere (Feldmann et al., 2012; Arndt et al., 2014b). Recombinant antibodies were produced by 3T3 cell lines (Feldmann et al., 2012; Arndt et al., 2014b). Antibody purification from cell culture supernatants was performed *via* Ni-NTA affinity chromatography (Feldmann et al., 2011). After dialysis of elution fractions against 1x PBS, proteins were characterized *via* SDS-PAGE and immunoblotting as published previously (Feldmann et al., 2011; Feldmann et al., 2012; Arndt et al., 2018).

### 2.3 Immunomagnetic isolation of CD3+ T cells

The study was approved by the local institutional review board of the Faculty of Medicine of the TU Dresden (EK138042014). Peripheral blood mononuclear cells (PBMCs) were isolated from buffy coats of healthy donors *via* density gradient centrifugation. Untouched CD3+ T cells were isolated from freshly prepared PBMCs using immunomagnetic separation according to the manufacturer’s instructions (Miltenyi Biotec GmbH, Bergisch Gladbach, Germany). The purity of the isolated cell population was > 90% as assessed by flow cytometric analysis. Isolated T cells were cultured in RPMI complete medium (Feldmann et al., 2011) supplemented with 50 U/ml interleukin (IL)-2 (Miltenyi Biotec GmbH).

### 2.4 Genetic modification of T cells

Generation of UniCAR T cells was carried out as described recently (Feldmann et al., 2020). Briefly, T cells were stimulated with T Cell TransAct™ (Miltenyi Biotec GmbH) and genetically modified *via* lentiviral transduction (Cartellieri et al., 2014) using a multiplicity of infection of 1–2. During transduction and expansion, T cells were maintained in TexMACS™ medium (Miltenyi Biotec GmbH) supplemented with human IL-2, human IL-7 and human IL-15 (all Miltenyi Biotec GmbH). Experiments were conducted with unsorted UniCAR T cells that were kept in RPMI complete medium (Feldmann et al., 2011) without additional cytokines for 24 h. Based on the co-translated EGFP marker protein expression, the proportion of UniCAR+ T cells was assessed *via* flow cytometry prior to each experiment.

### 2.5 Flow cytometric analysis

Analysis of surface molecules on CD3+ T cells was performed using the following monoclonal antibodies: APC-H7-conjugated anti-human CD3 (BD Biosciences, Heidelberg, Germany), anti-human CD4-VioBlue, anti-human CD3-FITC and anti-human CD8-APC (all Miltenyi Biotec GmbH). Immunofluorescence staining of cell surface molecules was performed using the relevant antibodies according to the provider’s instructions. After the staining procedure, cells were washed and evaluated by BD LSRFortessa™ flow cytometer or MACSQuant Analyzer 10 (Miltenyi Biotec GmbH). Before measurement, 7-AAD (BD Biosciences), DAPI (Miltenyi Biotec GmbH) or propidium iodide solution (Thermo Fisher Scientific, Waltham, Massachusetts, USA) was added for live/dead discrimination.

### 2.6 T cell proliferation

In order to distinguish effector and target cells, CD3+ T cells and freshly prepared or thawed UniCAR T cells were stained with cell proliferation dye eFluor™ 670 according to the manufacturer’s instructions (Thermo Fisher Scientific). Stained CD3+ T cells (2 × 10^5^/well) were cultured in the presence of stimulating anti-CD3/CD28 beads (Thermo Fisher Scientific) as well as the presence or absence of palbociclib (1, 0.2 or 0.025 µM) and fulvestrant (0.1 or 0.025 µM) in different concentrations in round-bottomed 96-well plates. Palbociclib was added daily whereas fulvestrant was added only once to the corresponding wells, according to the clinical dosing schedule. Cells were harvested after 24, 48, 72, 96, 120, 144 or 168 h. After excluding doublets and distinguishing between live and dead cells, the percentage of eFluor670+-diminished T cells compared to the untreated control was analyzed and expressed as “% proliferation”. Additionally, number of T cells were determined over time by gating on single, living eFluor670+ cells. Samples were analyzed after DAPI staining by utilizing a MACSQuant VYB flow cytometer (Miltenyi Biotec GmbH). For analysis of reversibility, palbociclib was only added at the first day and cells were harvested after 48 or 120 h and stained with anti-CD3 antibody. Percentage of eFluor™ 670 diminished CD3+ T cells was monitored by a BD LSRFortessa™ flow cytometer.

eFluor™ 670 stained unstimulated or UniCAR-modified T cells were incubated with tumor cells at an effector-to-target cell (E:T) ratio of 5:1 in the presence or absence of 30 nM CD3-PSCA bsAb or PSCA TM in a 96 h co-cultivation assay. Palbociclib (1, 0.2 or 0.025 µM) was added after 0, 24, 48 and 72 h. Fulvestrant (0.1 or 0.025 µM) was added just once at the beginning of the experiment (0 h). Effector cell numbers were determined by flow cytometry after 96 h as previously published (Arndt et al., 2020b). In brief, 20 µl of each sample was transferred to a 96-well round bottom plate and mixed with DAPI solution prior to measurement with the MACSQuant VYB analyzer (Miltenyi Biotec GmbH). Samples were first gated for T cells using SSC-A/FSC-A parameters. Subsequently, doublets were excluded by FSC-H/FSC-A gating and live/dead cells were distinguished by DAPI. Gating on eFluor670+ or eFluor670+ EGFP+ cells identified T cells or UniCAR T cells and allowed determination of T cell number.

### 2.7 EdU flow cytometry assay

To analyze DNA replication in proliferating cells the Click-iT^®^ Plus EdU Flow Cytometry Assay Kit was utilized according to the manufacturer’s instructions (Thermo Fisher Scientific). During DNA synthesis, the thymidine analog EdU (5-ethynyl-2′-deoxyuridine) was incorporated in the DNA and detected by flow cytometry after performing a copper catalyzed click reaction with the provided Alexa Fluor 488 dye. In brief, CD3+ T cells of four healthy donors (2 × 10^5^/well), being prepared as described in Section 2.3, were stimulated with human T-cell activator CD3/CD28 Dynabeads™ (Thermo Fisher Scientific) and cultured in the presence or absence of palbociclib at different concentrations (0.025, 0.2 or 1 µM) in round-bottomed 96-well plates. Unstimulated, untreated as well as stimulated and DMSO treated cells served as controls. Palbociclib was added daily during the experimental period of 4 days. 18 h prior to cell harvesting, cells were incubated with 10 µM EdU. The cells were harvested after 96 h and stained with an APC-coupled anti-CD3 antibody (BD Biosciences). Following surface antibody staining, cells were fixed and permeabilized using Click-iT^®^ fixative and 1x Click-iT^®^ saponin-based reagent, respectively, for 15 min each. Further, Click-iT^®^ Plus reaction cocktail was added and the samples were incubated, protected from light, for another 30 min at room temperature. After staining and washing procedure, samples were incubated with DAPI (0.4 ng/μl) to stain the cells for DNA content. Finally, flow cytometric analysis was performed using MACSQuant Analyzer 10 (Miltenyi Biotec GmbH) with the appropriate laser and filter settings. FlowLogic™ software (version 8.6; Inivai Technologies, Mentone Victoria, Australia) was used for data evaluation.

### 2.8 Cytokine assay

To evaluate the interferon (IFN)-γ and tumor necrosis factor (TNF) secretion of activated T cells, 5 × 10^4^ effector cells (unstimulated T cells or UniCAR T cells) and 1 × 10^4^ tumor cells were incubated in the presence or absence of 30 nM of recombinant antibody (CD3-PSCA bsAb or PSCA TM) in round-bottomed 96-well plates. Palbociclib (1, 0.2 or 0.025 µM) was added after 0, 24, 48 and 72 h. Fulvestrant (0.1 or 0.025 µM) was added just once at the beginning of the experiment (0 h). After 96 h, cell-free supernatants were collected. IFN-γ and TNF were quantified by ELISA according to the manufacturer’s instructions (BD Biosciences).

### 2.9 Luminescence-based killing assay

Luminescence-based killing assays were performed according to a previously published protocol (Mitwasi et al., 2017). Briefly, 5 × 10^4^ effector cells (unstimulated T cells or UniCAR T cells) and 1 × 10^4^ luciferase-expressing tumor cells were incubated with or without 30 nM of recombinant antibody (CD3-PSCA bsAb or PSCA TM). In addition, palbociclib (1, 0.2 or 0.025 µM) and/or fulvestrant (0.1 or 0.025 µM) were added to co-cultures. Alternatively, effector cells were first incubated with palbociclib (0.2 or 0.025 µM) and/or fulvestrant (0.1 or 0.025 µM) for 24 h. Thereafter, tumor cells and recombinant antibodies were added as described above. In addition, co-cultivation assays were repeatedly supplemented with palbociclib (1, 0.2 or 0.025 µM). Effector cells were prepared freshly or thawed 48 h prior to experiment. After 8 h of co-culture, 96-well white plates were centrifuged for 5 min at 360 × g. Subsequently, 100 μl supernatant was carefully removed and 50 μl ONE-Glo™ Luciferase reagent (Promega GmbH, Mannheim, Germany) added to each well. Following a 5 min incubation step, luminescence of each sample was measured using the Infinite^®^ M200 pro microplate reader (Tecan Germany GmbH, Crailsheim, Germany). Specific tumor cell lysis was calculated as described elsewhere (Mitwasi et al., 2017).

### 2.10 Statistical analysis

Student’s *t*-test was performed to evaluate the significance of the results. To compare samples to a control sample One-way ANOVA with posthoc Dunnett multiple comparison test was performed using GraphPad Prism 7 software (GraphPad Prism Inc., La Jolla, CA, United States). Values of *p* ≤ 0.05 were considered as significant.

## 3 Results

### 3.1 Palbociclib reversibly inhibits proliferation of stimulated CD3+ T cells

**FIGURE 1:**
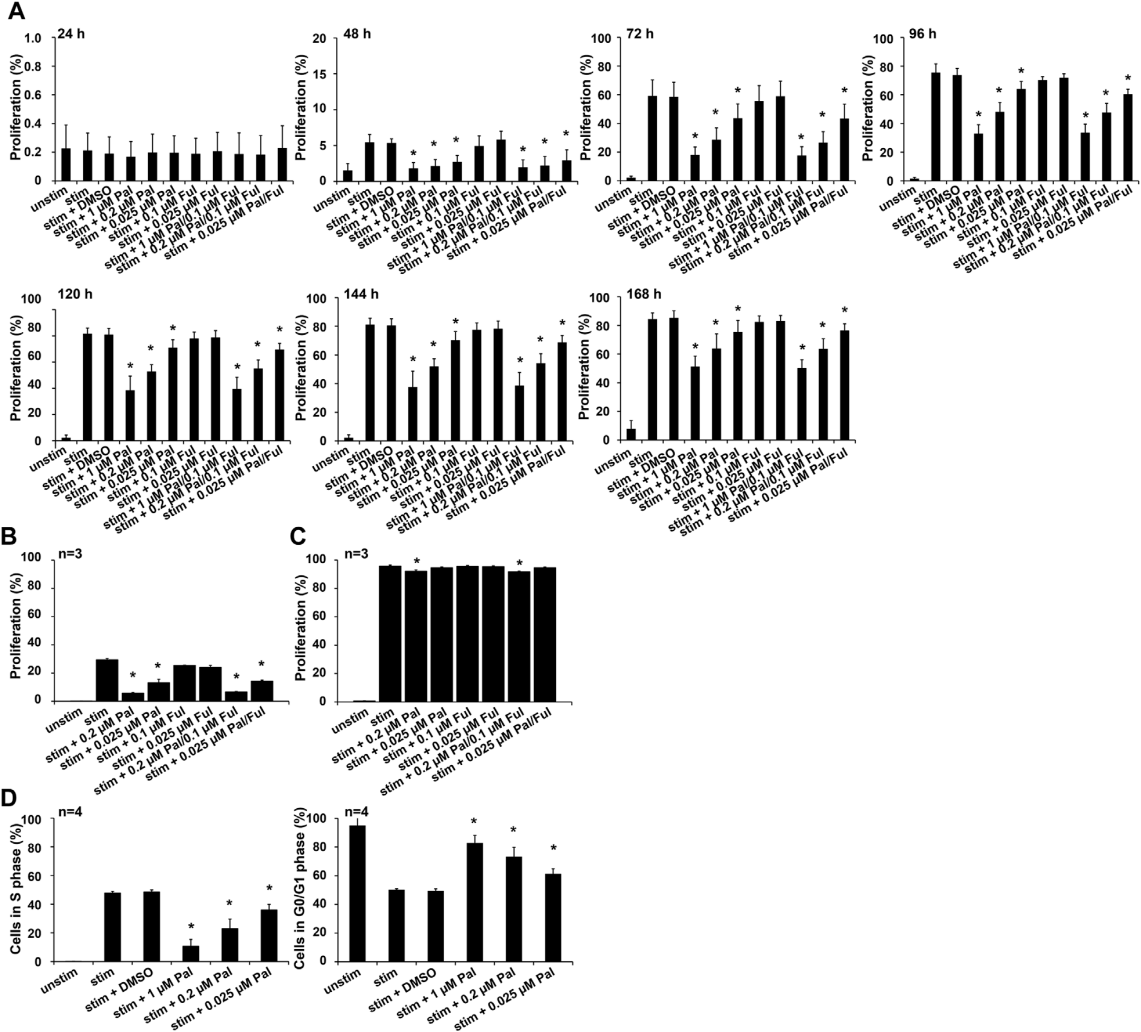
Influence of palbociclib and fulvestrant on T cell proliferation. **(A)** eFluor™ 670-stained T cells were stimulated by anti-CD3/CD28 beads and cultured in the presence or absence of palbociclib (daily addition), fulvestrant, or their combination at indicated concentrations for 24, 48, 72, 96, 120, 144, and 168 h. Cells were harvested and dilution of eFluor670™ dye was determined by flow cytometry. The results are depicted as the means ± SEM of four donors. (*p ≤ 0.05 compared to control sample “stim + DMSO”). **(B,C)** eFluor™ 670-stained T cells were stimulated by anti-CD3/CD28 beads and cultured in the presence or absence of palbociclib (single administration), fulvestrant, or their combination for **(B)** 48 h and **(C)** 120 h. Cells were harvested and dilution of proliferation dye eFluor670™ was determined by flow cytometry. The results are depicted as the means ± SEM of three donors. (*p ≤ 0.05 compared to control sample “stim”). **(D)** DNA replication in proliferating cells was analyzed by EdU flow cytometry assay. Therefore, T cells were stimulated by anti-CD3/CD28 beads and cultured in the presence of palbociclib (daily addition) at indicated concentrations for 96 h. 18 h prior to cell harvesting, cells were incubated with 10 µM EdU. Cells were harvested and percentage of EdU-positive cells was determined by flow cytometry. (*p ≤ 0.05 compared to control sample “stim + DMSO”).

The expansion, cytokine secretion and cytotoxic activity of T cells play an important role in antitumor immunity. Due to the fact, that T cells rapidly proliferate after antigen-specific activation, we explored the effect of the CDK4/6 inhibitor on T cell proliferation. To investigate whether palbociclib and/or fulvestrant alter this function, CD3+ T cells were maintained in the presence or absence of palbociclib or fulvestrant alone or in combination. T cells were stimulated to proliferate by anti-CD3/CD28 beads. Proliferation was measured every 24 h for 7 days by flow cytometry on the basis of eFluor™ 670 dilution over time. As depicted in Figure 1A, T cells displayed a strong proliferation upon stimulation in comparison to unstimulated control starting after 48 h. Palbociclib significantly impaired this ability of the CD3+ T cells in a concentration-dependent manner. Notably, 0.025–1 µM palbociclib clearly reduced the proliferative capacity of CD3+ T cells. The reduced proliferation, as measured by the dilution of the proliferation dye eFluor670™, is also reflected in a reduced number of T cells (Supplementary Figure S1). In contrast, fulvestrant did not influence this functional property of T cells (Figure 1A; Supplementary Figure S1).

We further investigated whether the inhibitory effect of palbociclib on T cell proliferation is reversible. Therefore, the CDK4/6 inhibitor was only added at the first day to stimulated CD3+ T cells instead of daily. Proliferation was analyzed after 48 h and 120 h. Although overall proliferation of stimulated T cells was less pronounced after 48 h, single-dosing of palbociclib alone or in combination with fulvestrant clearly impaired proliferation of CD3+ T cells (Figure 1B). However, after 120 h the inhibitory effect of palbociclib on T cell proliferation was almost abrogated (Figure 1C). These results indicate that the inhibitory effect of palbociclib on the proliferative capacity of T cells is reversible after stopping the drug application.

To study the underlying mechanisms of the palbociclib-mediated reduction in T cell proliferation, we performed an EdU assay with anti-CD3/CD28 bead-activated T cells treated with palbociclib (daily addition) for 96 h. We found a decreased percentage of T lymphocytes in S-phase of cell cycle when cells are exposed to 0.2 or 1 µM palbociclib (Figure 1D). In contrast, the percentage of T cells in G0/G1 phase increased under the same culture conditions. These findings indicate that the reduced proliferation of T cells is caused by a palbociclib-mediated G0/G1 cell cycle arrest.

### 3.2 Effect of palbociclib and fulvestrant on T cell-based immunotherapies

**FIGURE 2:**
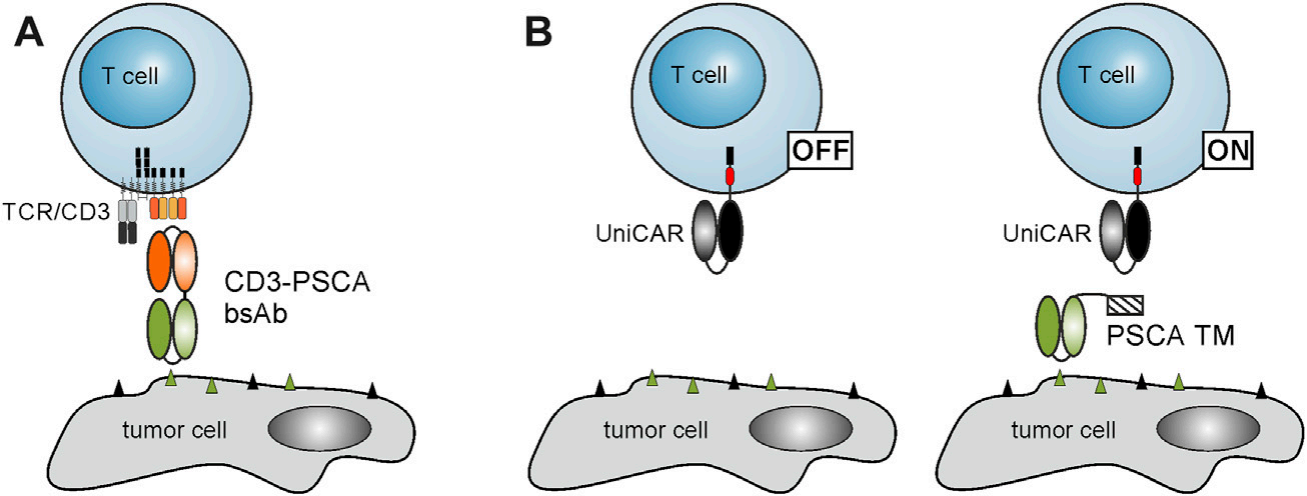
Schematic representation of T cell-based immunotherapies. **(A)** Due to its dual specificity for CD3 and PSCA, the CD3-PSCA bsAb is able to cross-link T cells and PSCA-expressing tumor cells. Subsequently, T cells are activated and kill the recognized target cell. **(B)** The UniCAR system is composed of UniCAR T cells and TAA-specific TMs. In the absence of TMs, UniCAR T cells are not activated. Upon addition of a PSCA-specific TM, UniCAR T cells can be cross-linked with PSCA-expressing tumor cells resulting in an efficient tumor cell lysis.

In view of potential combination therapies, we explored the influence of palbociclib and fulvestrant on two PSCA-specific T cell-based immunotherapies. On the one hand, we selected the CD3-PSCA bsAb (Figure 2A) (Feldmann et al., 2012). As shown in previous studies, due to its dual specificity for CD3 and PSCA it can specifically cross-link T cells and PSCA-expressing tumor cells in a MHC- and TCR-independent manner that finally culminates in effective tumor cell elimination (Feldmann et al., 2012). On the other hand, we chose the switchable UniCAR T cell technology (Bachmann, 2019), which is composed of UniCAR-modified T cells and PSCA-specific TMs (Figure 2B) (Arndt et al., 2014a; Arndt et al., 2014b).

In order to assess the impact of palbociclib and fulvestrant on both T cell retargeting strategies, co-cultivation assays with two different prostate cancer cells lines were carried out. For this purpose, unstimulated T cells or UniCAR T cells in the presence or absence of the CD3-PSCA bsAb or the PSCA TM were used. Palbociclib and fulvestrant were added either alone or in combination at various concentrations.

#### 3.2.1 Palbociclib markedly impairs the proliferative capacity of bsAb-engaged T cells and UniCAR T cells

**FIGURE 3:**
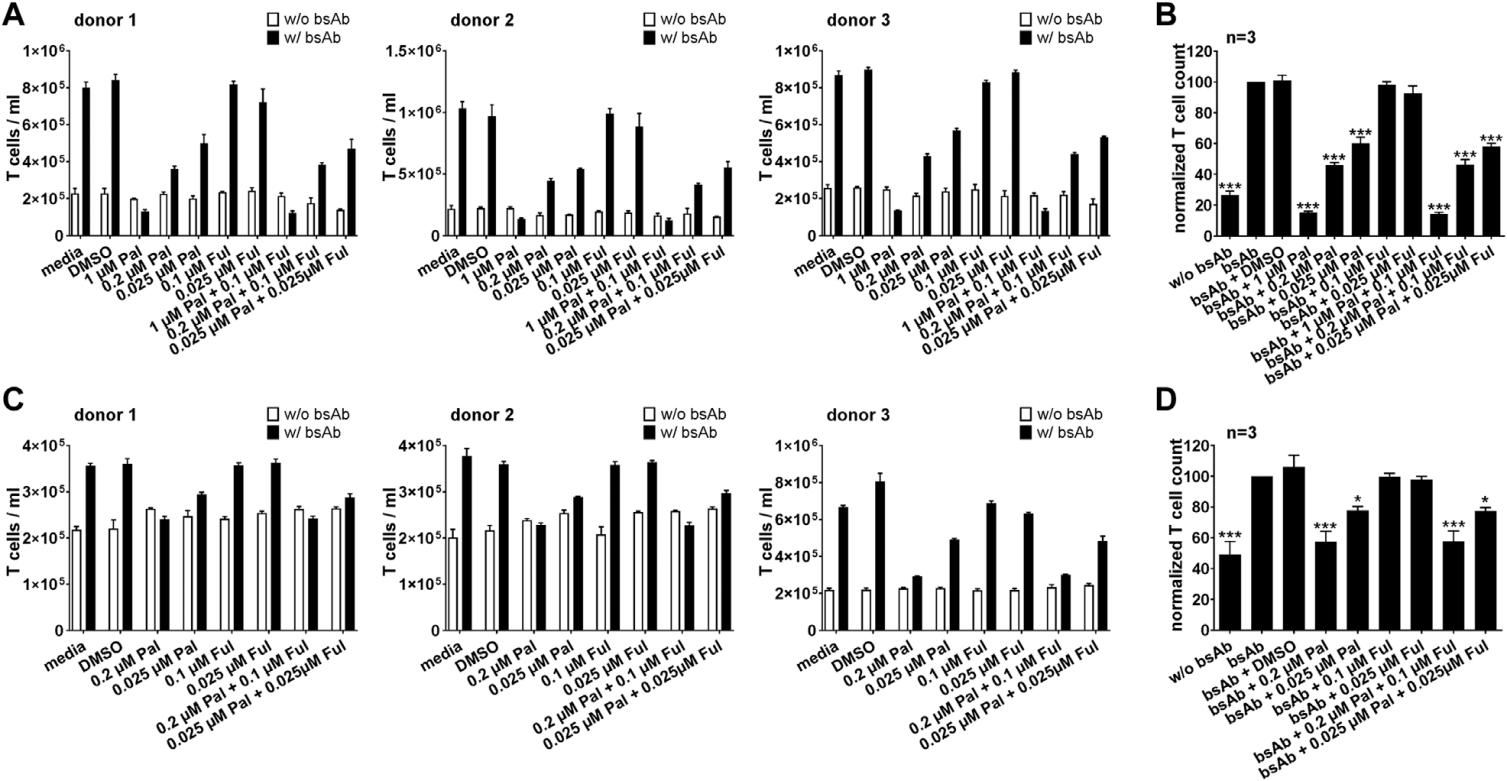
Effect of palbociclib and fulvestrant on bsAb-mediated T cell expansion. eFluor™ 670^+^ T cells and **(A,B)** PC3-PSCA/PSMA Luc+ or **(C,D)** LNCaP-PSCA Luc+ cells were incubated with or without CD3-PSCA bsAb. Palbociclib and/or fulvestrant were added at indicated concentrations. After 96 h, numbers of eFluor™ 670^+^ T cells were determined by flow cytometry using the MACSQuant^®^ Analyzer. **(A,C)** Each diagram shows average T cell counts ± SEM of triplicates for one T cell donor. **(B,D)** Graphs summarize relative T cell counts ± SEM of three different T cell donors. T cell numbers in the presence of tumor cells and bsAb (“bsAb”) were equalized to 100%. (*p ≤ 0.05, ***p ≤ 0.001 compared to control sample “bsAb”; One-way ANOVA with posthoc Dunnett multiple comparison test).

For a sustained and efficient antitumor response mediated by T cell-based immunotherapies, proliferation and polyclonal expansion of effector T cell populations is required. Thus, first experiments aimed to investigate the influence of palbociclib and fulvestrant on the proliferative capacity of specifically activated bsAb-engaged T cells and UniCAR T cells. As shown in Figure 3, upon cross-linkage with PC3-PSCA/PSMA Luc+ (Figures 3A, B) or LNCaP-PSCA Luc+ cells (Figures 3C, D) *via* the CD3-PSCA bsAb, T cell numbers increased up to 5-fold compared to the negative controls without bsAb after 96 h. However, in the presence of 0.2 or 1 µM palbociclib the proliferation and expansion of bsAb-redirected T cells was efficiently inhibited (Figure 3). Under these conditions, numbers of T cells did not or only slightly increase and were in some cases almost equal to T cell counts detected in samples without the CD3-PSCA bsAb. Similar results were obtained when palbociclib was applied in combination with 0.1 μM fulvestrant. Although suppressive effects of palbociclib on T cell proliferation were less profound at a lower concentration of 0.025 μM, T cell expansion was still significantly reduced compared to samples lacking the small-molecule inhibitor. In contrast, the selective ER degrader fulvestrant did not alter bsAb-mediated T cell expansion.

**FIGURE 4:**
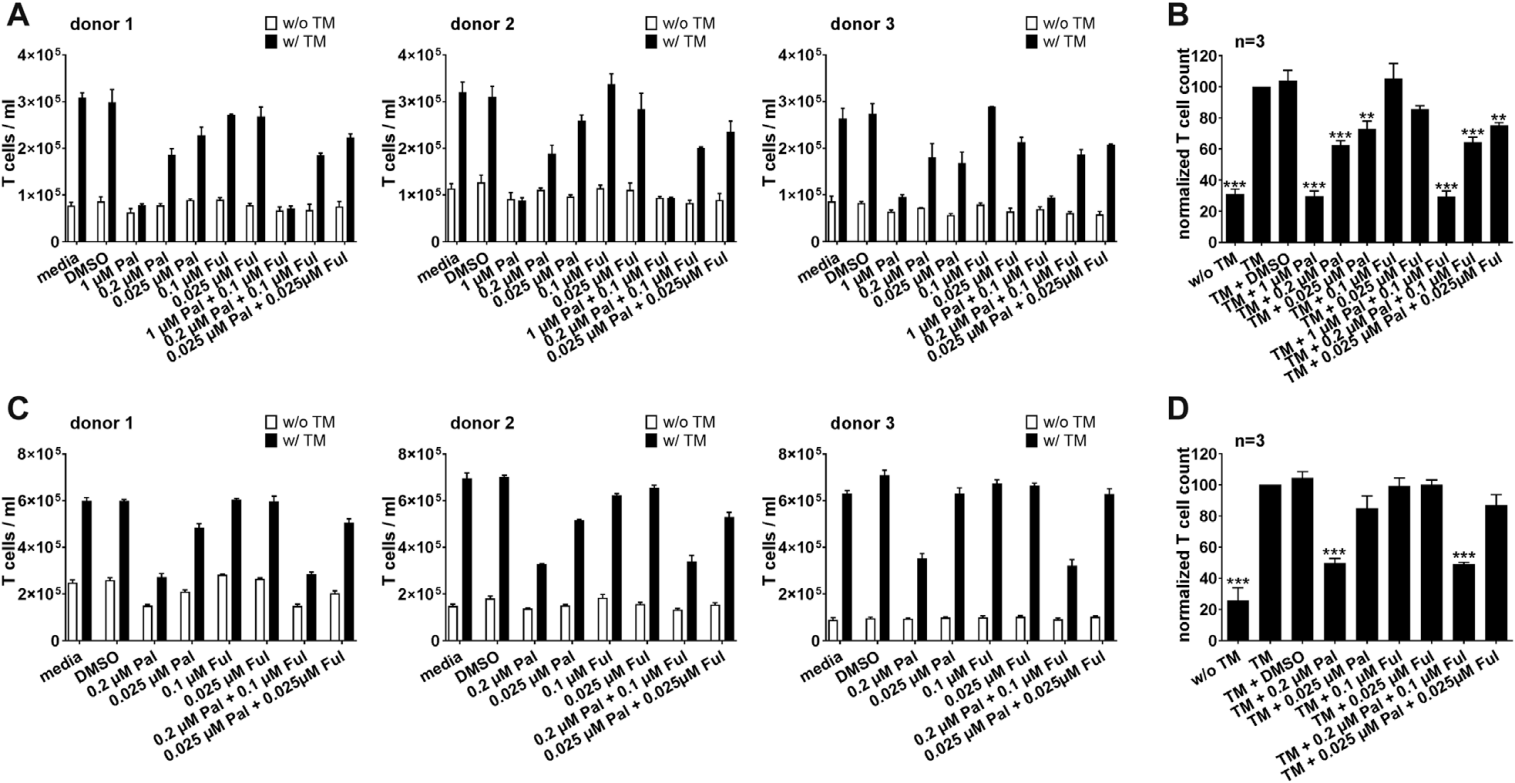
Effect of palbociclib and fulvestrant on TM-mediated UniCAR T cell expansion. eFluor™ 670^+^ UniCAR T cells and **(A,B)** PC3-PSCA/PSMA Luc+ or **(C,D)** LNCaP-PSCA Luc+ cells were incubated with or without 30 nM of PSCA TM. Palbociclib and/or fulvestrant were added at indicated concentrations. After 96 h, numbers of eFluor™ 670+ UniCAR T cells were determined by flow cytometry using the MACSQuant^®^ Analyzer. **(A,C)** Each diagram shows average T cell counts ± SEM of triplicates for one T cell donor. **(B,D)** Graphs summarize relative UniCAR T cell counts ± SEM of three different T cell donors. UniCAR T cell numbers in the presence of tumor cells and TM (“TM”) were equalized to 100%. (***p ≤ 0.001 compared to control sample “TM”; One-way ANOVA with posthoc Dunnett multiple comparison test).

These findings were not only limited to bsAb-redirected T cells, but could also be observed when palbociclib and fulvestrant were combined with the PSCA-specific UniCAR system. Expansion of redirected UniCAR T cells was significantly suppressed in the presence of 0.2 or 1 µM palbociclib alone or in combination with 0.1 μM fulvestrant (Figure 4). Fulvestrant alone exerted no inhibitory effects on the proliferative capacity of UniCAR T cells.

#### 3.2.2 Impact of palbociclib on IFN-γ and TNF secretion by bsAb-engaged T cells and UniCAR T cells

**FIGURE 5:**
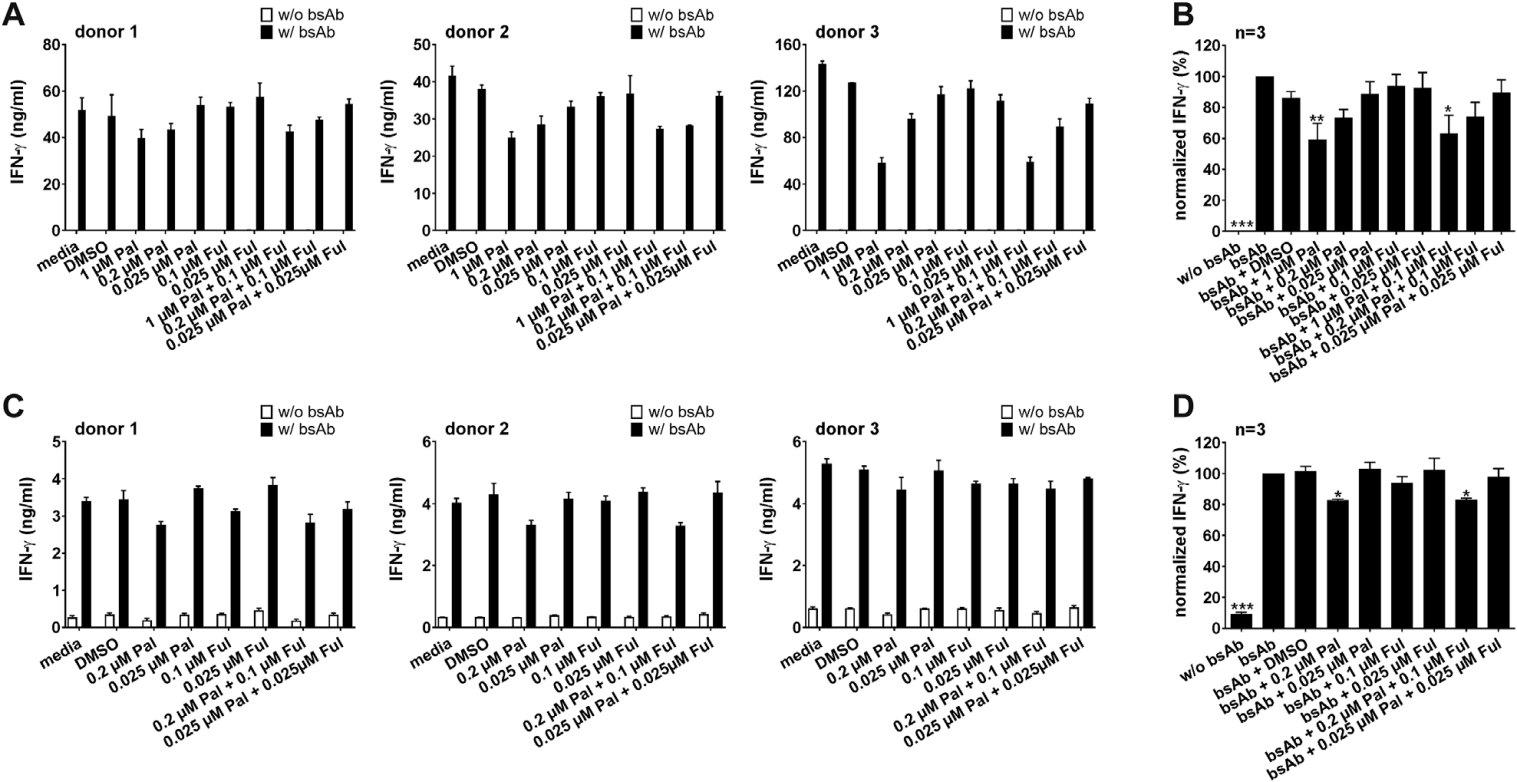
Effect of palbociclib and fulvestrant on IFN-γ release of bsAb-engaged T cells. T cells and **(A,B)** PC3-PSCA/PSMA Luc+ or **(C,D)** LNCaP-PSCA Luc+ cells were incubated with or without 30 nM of CD3-PSCA bsAb in the presence or absence of palbociclib and/or fulvestrant. After 96 h, IFN-γ concentrations in co-culture supernatants were analyzed by ELISA. **(A,C)** Each diagram shows average IFN-γ concentration ± SEM of triplicates for one T cell donor. **(B,D)** Graphs summarize relative IFN-γ release ± SEM of three different T cell donors. IFN-γ concentrations in the presence of T cells, tumor cells and bsAb (“bsAb”) were equalized to 100%. (*p ≤ 0.05, ***p ≤ 0.001 compared to control sample “bsAb”; One-way ANOVA with posthoc Dunnett multiple comparison test).

As the proinflammatory cytokines IFN-γ and TNF can increase the therapeutic efficacy of T cell-based immunotherapies, we investigated the impact of palbociclib and fulvestrant on the secretion of IFN-γ and TNF by UniCAR T cells and bsAb-redirected T cells. Therefore, cytokine assays were performed with LNCaP-PSCA Luc+ or PC3-PSCA/PSMA Luc+ cells. Upon cross-linkage with PC3-PSCA/PSMA Luc+ (Figures 5A, B) or LNCaP-PSCA Luc+ cells (Figures 5C, D) *via* the CD3-PSCA bsAb, a higher IFN-γ concentration in the supernatants compared to the control was detected. Palbociclib used at a concentration of 0.2 or 1 µM significantly reduced the IFN-γ concentration in the supernatants of bsAb-redirected T cells. A lower palbociclib concentration (0.025 µM) exerted only minor or no inhibitory effects. Likewise, in the presence of 1 µM palbociclib, significantly lower levels of TNF were detected in the co-culture supernatants after 96 h (Supplementary Figures S2A, B). Fulvestrant alone did not affect the bsAb-mediated IFN-γ or TNF secretion by T cells.

**FIGURE 6:**
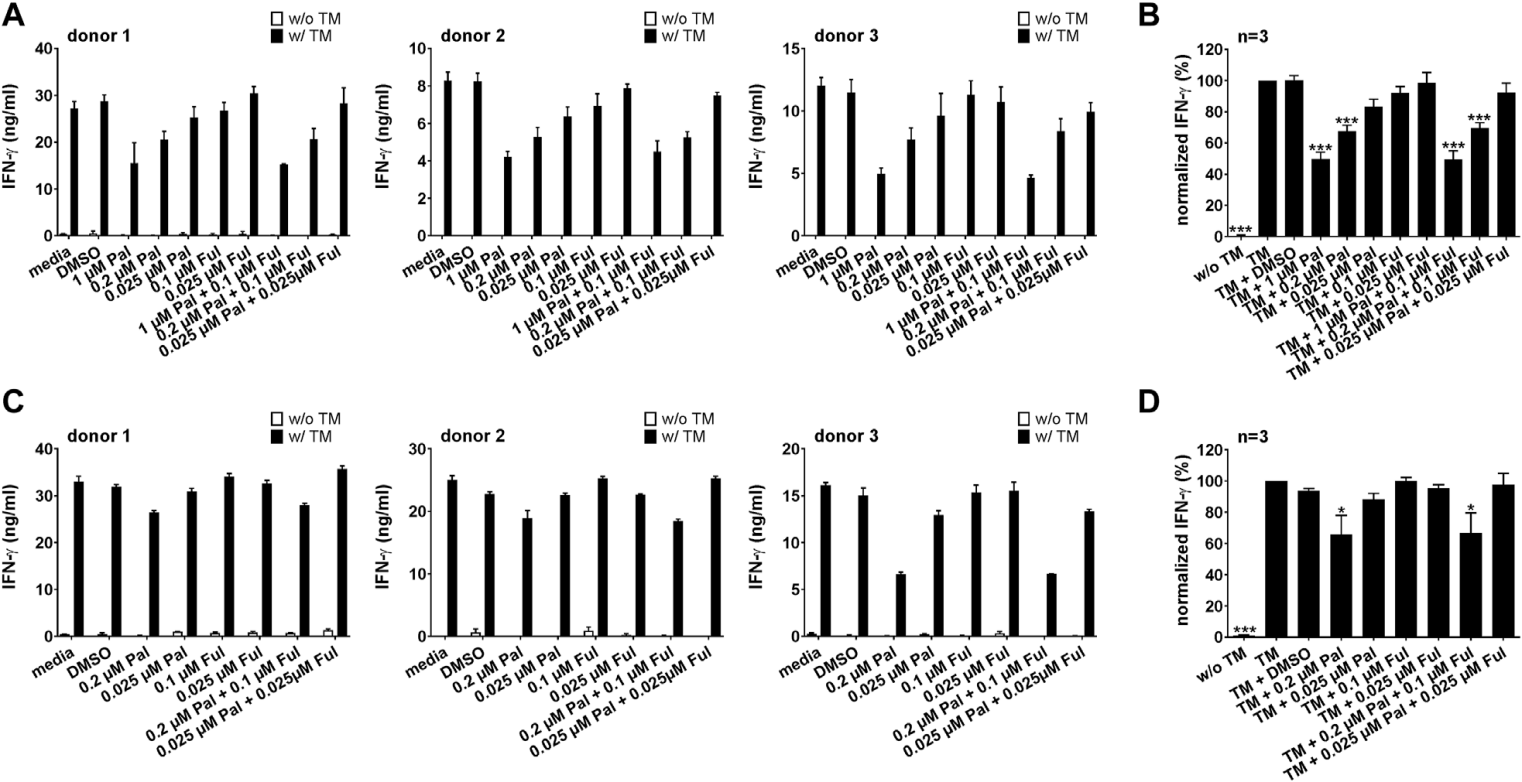
Effect of palbociclib and fulvestrant on IFN-γ release of TM-engaged UniCAR T cells. UniCAR T cells and **(A,B)** PC3-PSCA/PSMA Luc+ or **(C,D)** LNCaP-PSCA Luc+ cells were incubated with or without 30 nM of PSCA TM in the presence or absence of palbociclib and/or fulvestrant. After 96 h, IFN-γ concentrations in co-culture supernatants were analyzed by ELISA. **(A,C)** Each diagram shows average IFN-γ concentration ± SEM of triplicates for one T cell donor, respectively. **(B,D)** Graphs summarize relative IFN-γ release ± SEM of three different T cell donors. IFN-γ concentrations in the presence of UniCAR T cells, tumor cells and TM (“TM”) were equalized to 100%. (*p ≤ 0.05, **p ≤ 0.01, ***p ≤ 0.001 compared to control sample “TM”; One-way ANOVA with posthoc Dunnett multiple comparison test).

Similar results were observed when palbociclib and fulvestrant were combined with the PSCA-specific UniCAR system (Figure 6; Supplementary Figures S2C, D). Cross-linkage of PSCA-specific UniCAR T cells with prostate cancer cells resulted in a higher IFN-γ and TNF concentration in the supernatants compared to the control. In the presence of palbociclib, the concentration of IFN-γ and TNF in the supernatants of redirected UniCAR T cells was reduced in a concentration-dependent manner. While in the presence of 0.2 or 1 µM palbociclib IFN-γ and TNF were significantly reduced, a lower palbociclib concentration only considerably altered TNF release. In contrast, fulvestrant had no inhibitory effect on this functional characteristic.

To explore whether the lower total concentrations of IFN-γ and TNF in the supernatants are associated with the palbociclib-mediated reduction of the T cell number, the amounts of IFN-γ and TNF detected in the supernatant after 96 h were calculated per T cell or UniCAR T cell. As summarized in Supplementary Figures S3, S4, in the presence of palbociclib average cytokine concentrations per cell did not significantly change or were even elevated compared to the settings without inhibitors, indicating that the lower concentrations of IFN-γ and TNF in the supernatants are associated with the palbociclib-induced decrease in T cell numbers.

#### 3.2.3 Cytotoxic potential of bsAb-engaged T cells and UniCAR T cells is unaffected by palbociclib

**FIGURE 7:**
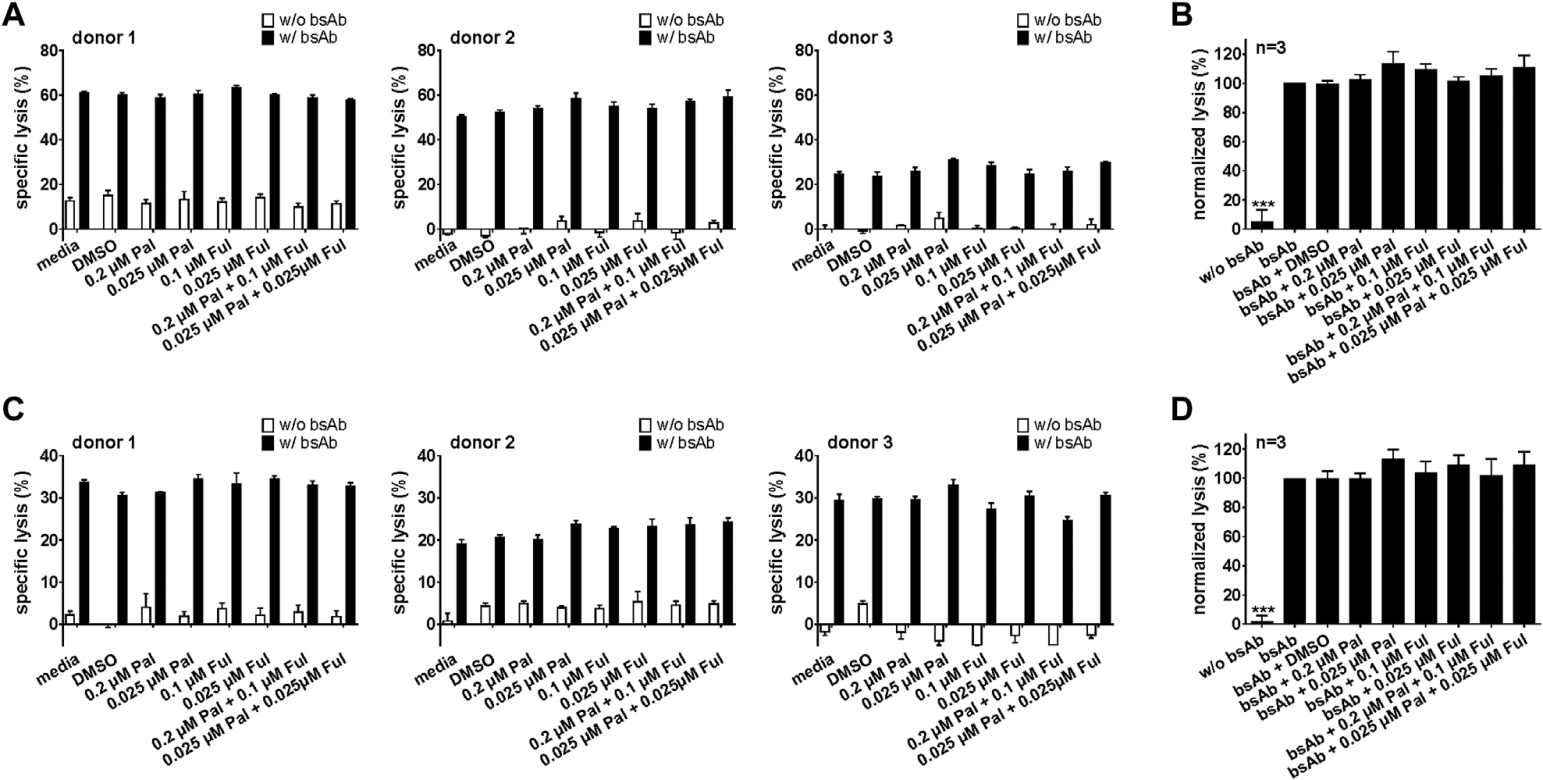
Effect of palbociclib and fulvestrant on bsAb-mediated tumor cell killing. T cells and **(A,B)** LNCaP-PSCA Luc+ or **(C,D)** PC3-PSCA/PSMA Luc+ cells were incubated with or without 30 nM of CD3-PSCA bsAb in the presence or absence of palbociclib and/or fulvestrant. After 8 h, tumor cell killing was calculated based on a luminescence-based killing assay. **(A,C)** Each diagram shows mean specific lysis ± SEM of triplicates for one T cell donor. **(B,D)** Graphs summarize relative tumor lysis ± SEM of three different T cell donors. Specific lysis in the presence of T cells and bsAb (“bsAb”) were equalized to 100%. (***p ≤ 0.001 compared to control sample “bsAb”; One-way ANOVA with posthoc Dunnett multiple comparison test).

For an effective combination therapy, it is important to ensure that palbociclib and fulvestrant do not impede antitumor cytotoxicity of T cell-based immunotherapies. Hence, the effect of palbociclib and fulvestrant on bsAb- or UniCAR T cell-mediated tumor cell killing was investigated by performing luminescence-based killing assays with LNCaP-PSCA Luc+ or PC3-PSCA/PSMA Luc+ cells. As shown in Figure 7 and Supplementary Figures S5A, 5B, the CD3-PSCA bsAb was able to specifically engage T cells for killing of PSCA+ tumor cells already after 8 h. Interestingly, palbociclib and/or fulvestrant did not modulate this functional property of T cells.

**FIGURE 8:**
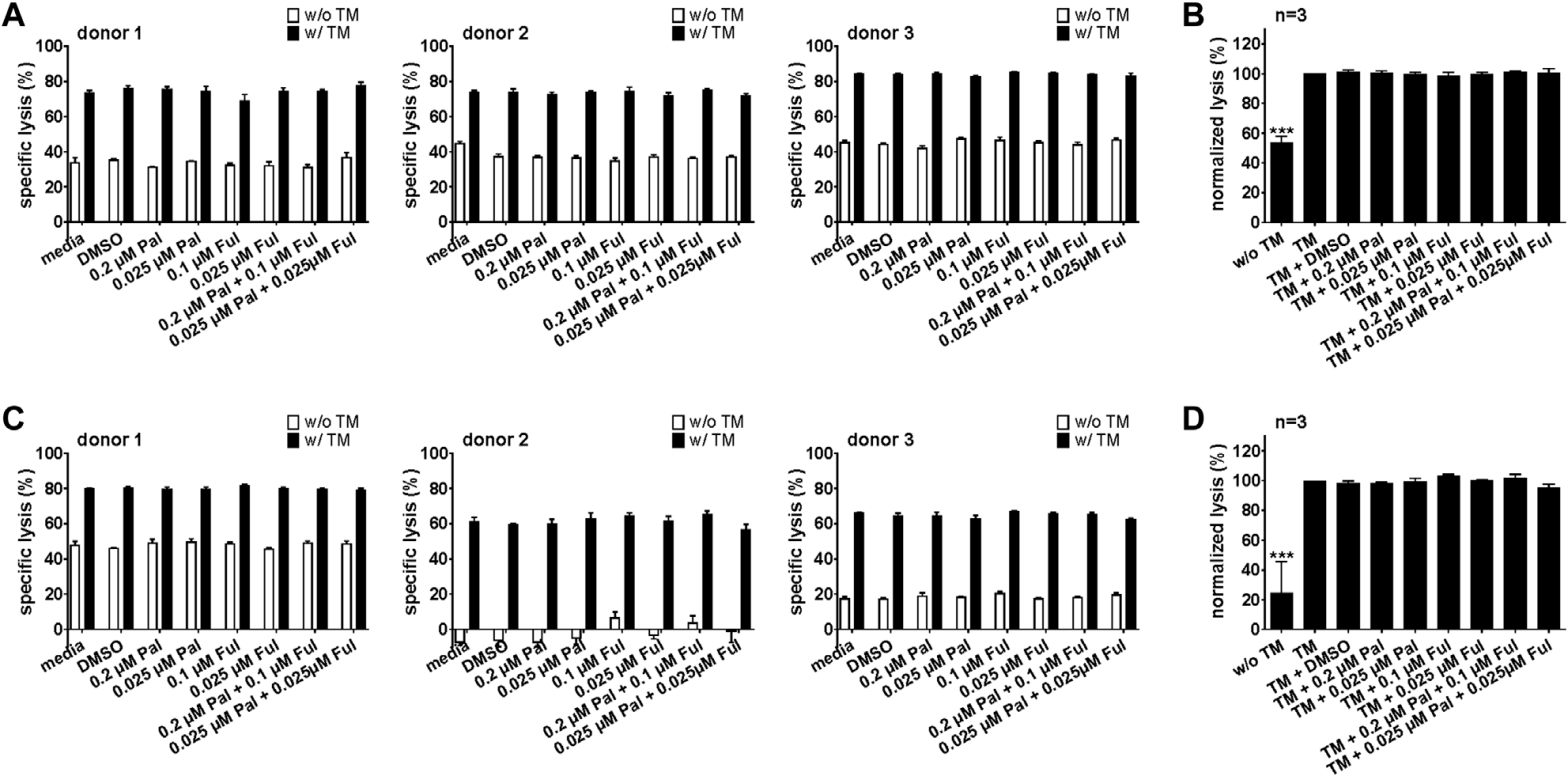
Effect of palbociclib and fulvestrant on UniCAR T cell-mediated tumor cell killing. UniCAR T cells and **(A,B)** LNCaP-PSCA Luc+ or **(C,D)** PC3-PSCA/PSMA Luc+ cells were incubated with or without 30 nM of PSCA TM in the presence or absence of palbociclib and/or fulvestrant. After 8 h, tumor cell killing was calculated based on a luminescence-based killing assay. **(A,C)** Each diagram shows mean specific lysis ± SEM of triplicates for one T cell donor. **(B,D)** Graphs summarize relative tumor lysis ± SEM of three different T cell donors. Specific lysis in the presence of UniCAR T cells and TM (“TM”) were equalized to 100%. (***p ≤ 0.001 compared to control sample “TM”; One-way ANOVA with posthoc Dunnett multiple comparison test).

Similar results were obtained for the PSCA-specific UniCAR system (Figure 8; Supplementary Figures S5C, D). Upon cross-linkage with prostate cancer cells *via* the PSCA TM, UniCAR T cells mediated efficient tumor cell lysis. Again, palbociclib and/or fulvestrant did not alter the cytotoxic activity of the UniCAR T cells.

**FIGURE 9:**
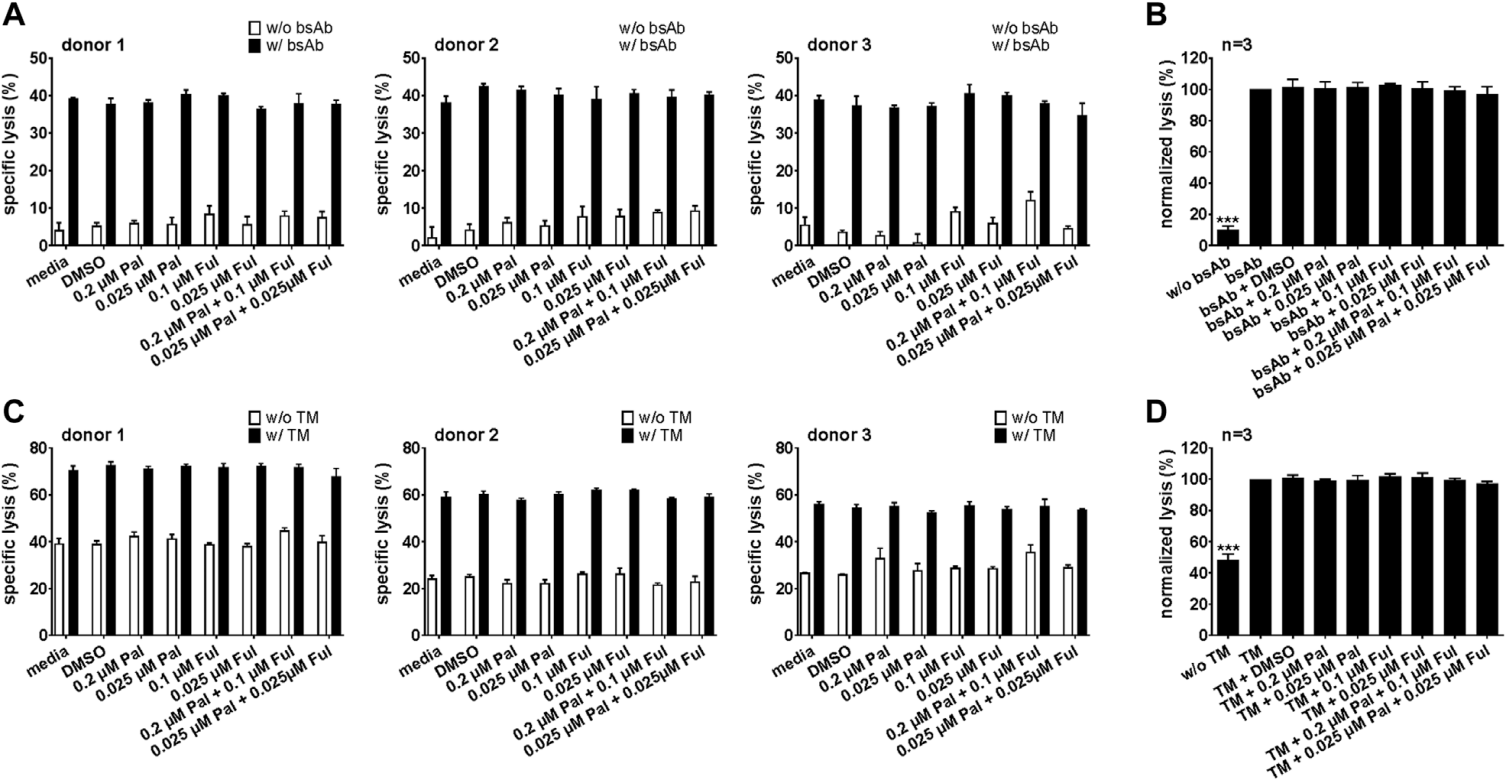
Cytotoxic capacity of palbociclib- and fulvestrant-pretreated bsAb-engaged T cells and UniCAR T cells. After pre-incubation of **(A,B)** T cells or **(C,D)** UniCAR T cells without or with palbociclib and/or fulvestrant for 24 h, luciferase-based killing assays were performed. Therefore, preincubated **(A,B)** T cells or **(C,D)** preincubated UniCAR T cells were cultured with PC3-PSCA/PSMA Luc+ cells in the presence or absence of 30 nM **(A,B)** CD3-PSCA bsAb or **(C,D)** PSCA TM. After 8 h, specific lysis was determined. **(A,C)** Each diagram shows mean specific lysis ± SEM of triplicates for one T cell donor. **(B,D)** Graphs summarize relative tumor lysis ± SEM of three different T cell donors. Specific lysis in the presence of **(B)** T cell and bsAb (“bsAb”) or **(D)** UniCAR T cells and TM (“TM”) were equalized to 100%. (***p ≤ 0.001 compared to control sample “bsAb” or “TM”; One-way ANOVA with posthoc Dunnett multiple comparison test).

In a next step, we also investigated whether pretreatment of T cells or UniCAR T cells with palbociclib and/or fulvestrant can influence their cytotoxic potential. For this purpose, T cells or UniCAR T cells were incubated with palbociclib and/or fulvestrant at 37°C. 24 h later, luminescence-based killing assays were performed in which drug-pretreated T cells or UniCAR T cells were co-cultured with PC3-PSCA/PSMA Luc+ cells and 30 nM CD3-PSCA bsAb or PSCA TM, respectively. Pre-incubation of T cells (Figures 9A,B) or UniCAR T cells (Figures 9C,D) with one or both drugs did neither influence tumor cell killing mediated by bsAb-redirected T cells nor by TM-engaged UniCAR T cells. These results provide evidence that palbociclib alone or in combination with fulvestrant retain the cytotoxic activity of bsAb-engaged T cells and UniCAR T cells.

## 4 Discussion

CDK4/6 inhibitors in combination with endocrine therapy received FDA approval for treatment of patients with ER+, HER2- advanced or metastatic breast cancer (Finn et al., 2016a; Cristofanilli et al., 2016; Hortobagyi et al., 2016). This kind of therapy has shown clinical efficacy by prolonging PFS and OS (Finn et al., 2016a; Cristofanilli et al., 2016; Hortobagyi et al., 2016; Im et al., 2019; Johnston et al., 2020; Slamon et al., 2020; Sledge et al., 2020). However, a proportion of patients experienced disease progression (Finn et al., 2016a; Finn et al., 2016b; Cristofanilli et al., 2016; Hortobagyi et al., 2016). Potential explanations for therapy resistance to CDK4/6 inhibition in these patients are intrinsic or acquired resistance, including deregulations of the immune pathway, such as activation of inhibitory immune checkpoint pathways and suppression of immune stimulatory pathways in palbociclib-resistant cells (Pandey et al., 2019; Pandey et al., 2021; Pancholi et al., 2020). Therefore, identification of novel therapeutic strategies for treatment of patients resistant to CDK4/6 inhibition is urgently needed. In the last few decades, immunotherapy has become an important treatment modality. Since functional T cells are a crucial component of the tumor microenvironment for efficient tumor eradication, they emerged as key players of diverse immunotherapeutic strategies, including inhibition of immune checkpoint molecules and adoptive cellular therapy. With regard to therapeutic combination strategies, first success was achieved combining palbociclib with immune checkpoint inhibitors (CPI) in an *ex vivo* organotypic tumor spheroid culture system as well as in different mouse models (Deng et al., 2018; Zhang et al., 2018; Long et al., 2020). Furthermore, the comparison of patients with metastatic breast cancer treated with a combination of palbociclib, the CPI pembrolizumab and the aromatase inhibitor letrozole or with pembrolizumab and letrozole revealed that higher frequencies of blood-circulating effector memory T cells at baseline are potential predictive biomarkers of response to the combination of CDK4/6 inhibitors and CPI (Egelston et al., 2021). Additional attractive T cell-based strategies, such as bsAb (Wolf et al., 2005) and CAR T cell therapy (June et al., 2018; Arndt et al., 2020a), may also represent promising combinatorial partners for CDK4/6 inhibitors.

Emerging preclinical studies revealed diverse immunomodulatory effects of CDK4/6 inhibition, such as an enhanced immune infiltration into the tumor microenvironment, elevated antigen presentation and modulation of the cytokine milieu, supporting antitumor immune response (Ameratunga et al., 2019). Different murine models investigated the influence of CDK4/6 inhibition on tumor-infiltrating immune cells. In a breast cancer mouse model, a significant reduction of CD3+ tumor-infiltrating lymphocytes was observed under palbociclib treatment (Zhang et al., 2018). In contrast, Goel and colleagues reported significant increases of intratumoral CD3+ T cells in their transgenic mouse model of mammary carcinoma treated with abemaciclib or palbociclib (Goel et al., 2017). Another study also showed enhanced proportions of CD4+ and CD8+ T cells in lung tumors of genetically engineered mice after treatment with palbociclib or trilaciclib (Deng et al., 2018). However, this effect seems not to be based on increased proliferation, but on elevated homing of effector T cells to the tumor. These data are in line with observations by Schaer and colleagues, who demonstrated that abemaciclib increased the frequency of CD3+ T cell numbers within the tumor, but not the absolute numbers in a colon cancer mouse model (Schaer et al., 2018). By analyzing the functional status of the tumor-infiltrating T cells, Deng et al. (2018) observed an increased IFN-γ secretion by total splenocytes isolated from lung tumor-bearing mice, but not naïve mice, treated with trilaciclib *in vivo*. Moreover, Teo and colleagues have shown, that the CDK4/6 inhibitor ribociclib does not impair activation and cytotoxic potential of tumor-infiltrating CD8+ and CD4+ T cells in a triple-negative breast cancer mouse model (Teo et al., 2017).

Whereas all these findings rely on mouse models, little is known about the impact of palbociclib and fulvestrant on the functional properties of human immune cells. Heckler and colleagues investigated the impact of CDK4/6 inhibitors on activated CD8+ T cells of breast cancer patients and observed that CDK4/6 inhibition resulted in an increased frequency of memory CD8+ T cell precursors (Heckler et al., 2021). Based on the observation that higher frequencies of pre-existing effector memory T cells were detectable in the blood of responders to the combination of CDK4/6 inhibitors and CPI (Egelston et al., 2021), early treatment with CDK4/6 inhibitors to establish a memory CD8+ T cell pool followed by the CPI administration may represent a promising therapeutic strategy for cancer patients. Here, we examined the influence of palbociclib and fulvestrant on the proliferation and functional properties of bsAb-engaged T cells and UniCAR T cells in terms of a potential combinatorial approach of CDK4/6 inhibition and T cell-based immunotherapy. An important prerequisite for a durable and efficient antitumor T cell response is the proliferation and clonal expansion of the T cells. Therefore, we analyzed the impact of palbociclib and fulvestrant on the proliferative capacity of stimulated CD3+ T cells. We demonstrated that palbociclib clearly impairs the ability of T cells to proliferate upon anti-CD3/CD28 bead stimulation. This effect is based on a G0/G1 cell cycle arrest mediated by palbociclib. The palbociclib-mediated T cell inhibition was reversible after stopping the drug application. In contrast, fulvestrant did not influence this functional property. Furthermore, we analyzed the impact of palbociclib and fulvestrant on the proliferation of bsAb-engaged T cells and UniCAR T cells. Our studies were performed exemplarily with the CD3-PSCA bsAb (Feldmann et al., 2012) and the UniCAR technology using the PSCA TM (Arndt et al., 2014a; Arndt et al., 2014b; Bachmann, 2019). PSCA is a TAA upregulated in several major cancers, including prostate cancer, urinary bladder cancer, renal cell carcinoma, pancreatic cancer, ovarian mucinous tumor and NSCLC (Amara et al., 2001; Argani et al., 2001; Cao et al., 2005; Elsamman et al., 2006; Kawaguchi et al., 2010). Recently, PSCA expression in a subgroup of breast cancer patients was also reported (Link et al., 2017). Although T cell stimulation occurred *via* different methods, palbociclib but not fulvestrant prevented adequate proliferation and expansion of both bsAb-engaged T cells and redirected UniCAR T cells. Goel et al. also observed an inhibitory effect on the proliferation of CD4+ CD25- and CD8+ T cells derived from spleens and lymph nodes of wild-type mice after treatment with abemaciclib *in vitro*, concordant with our findings (Goel et al., 2017). Furthermore, *ex vivo* stimulation of splenocytes from lung tumor-bearing mice showed a reduced proliferation after anti-CD3/CD28 antibody stimulation and treatment with trilaciclib (Deng et al., 2018). In further experiments, we investigated the impact of palbociclib and/or fulvestrant on the cytokine production of T cells. The proinflammatory cytokine IFN-γ is an important player in the antitumoral immune response and may further enhance therapeutic effects of a T cell-based immunotherapy as it increases MHC class I expression and antigen presentation of tumor cells and further supports macrophages, cytotoxic T lymphocytes and natural killer cells in their antitumor response. TNF can also exhibit various antitumor effects, such as the induction of tumor cell apoptosis, and the recruitment and activation of tumor-reactive T cells. Palbociclib treatment of PSCA-specific UniCAR T cells and CD3-PSCA bsAb-engaged T cells co-cultured with prostate cancer cells resulted in lower total concentrations of IFN-γ and TNF in the supernatants. When investigating the underlying mechanism for this observation, we found that the lower concentrations of IFN-γ and TNF are linked to the palbociclib-mediated decrease in T cell numbers. For efficient tumor eradication, cytotoxic potential of T cells is a crucial parameter for immunotherapeutic strategies. For this reason, we further investigated the impact of palbociclib and fulvestrant on the UniCAR T cell- or bsAb-mediated cytotoxicity. The CD3-PSCA bsAb as well as the PSCA-specific UniCAR system have proven their capability to redirect T cells for efficient killing of PSCA+ tumor cells *in vitro* and in mouse models (Feldmann et al., 2012; Feldmann et al., 2017; Pishali Bejestani et al., 2017). We demonstrated under various conditions that neither palbociclib nor fulvestrant added either prior or during co-culture influence this functional property.

In summary, our data revealed that palbociclib reversibly impairs proliferation of activated CD3+ T cells and reduces expansion of UniCAR T cells and bsAb-engaged T cells. Furthermore, we observed reduced amounts of IFN-γ and TNF in the supernatants of palbociclib-treated PSCA-specific UniCAR T cells and CD3-PSCA bsAb-engaged T cells co-cultured with prostate cancer cells, which is caused by the palbociclib-mediated decrease in T cell numbers. The cytotoxic potential of PSCA-specific UniCAR T cells and CD3-PSCA bsAb-engaged T cells was not affected by palbociclib. Fulvestrant did not show impact on any of these functional properties of stimulated CD3+ T cells, UniCAR T cells and bsAb-engaged T cells. These results provide evidence that the CDK4/6 inhibitor palbociclib has not only an impact on the cell cycle of tumor cells but also on T cells, which are crucial players in an antitumor immune response. Hence, palbociclib-mediated alterations of T cell functionality should be taken into consideration for future therapeutic recommendations and potential combinatorial approaches with T cell-based immunotherapy. Thus, a palbociclib-free time period may be required for an efficient T cell-based immunotherapy.

## Data Availability

The original contributions presented in the study are included in the article/Supplementary Material, further inquiries can be directed to the corresponding authors.
