# Supplementary material for: Palbociclib impairs the proliferative capacity of activated T cells while retaining their cytotoxic efficacy
Source: Front Pharmacol. 2023 Feb 3;14:970457. doi: 10.3389/fphar.2023.970457 (PMC9935825; doi:10.3389/fphar.2023.970457)
Supplement: Supplementary file 1 [file DataSheet1.docx]

Supplementary Material

Palbociclib impairs the proliferative capacity of activated T cells while retaining their cytotoxic efficacy

Claudia Arndt*, Antje Tunger, Rebekka Wehner, Rebecca Rothe, Eleni Kourtellari, Stephanie Luttosch, Katharina Hannemann, Stefanie Koristka, Liliana R. Loureiro, Anja Feldmann, Torsten Tonn, Theresa Link, Jan Dominik Kuhlmann, Pauline Wimberger, Michael Philipp Bachmann and Marc Schmitz*

*** Correspondence:** Claudia Arndt: [c.arndt@hzdr.de](mailto:c.arndt@hzdr.de); Marc Schmitz: [marc.schmitz@tu-dresden.de](mailto:marc.schmitz@tu-dresden.de)

# Supplementary Figures and Tables

## Supplementary Figures

**
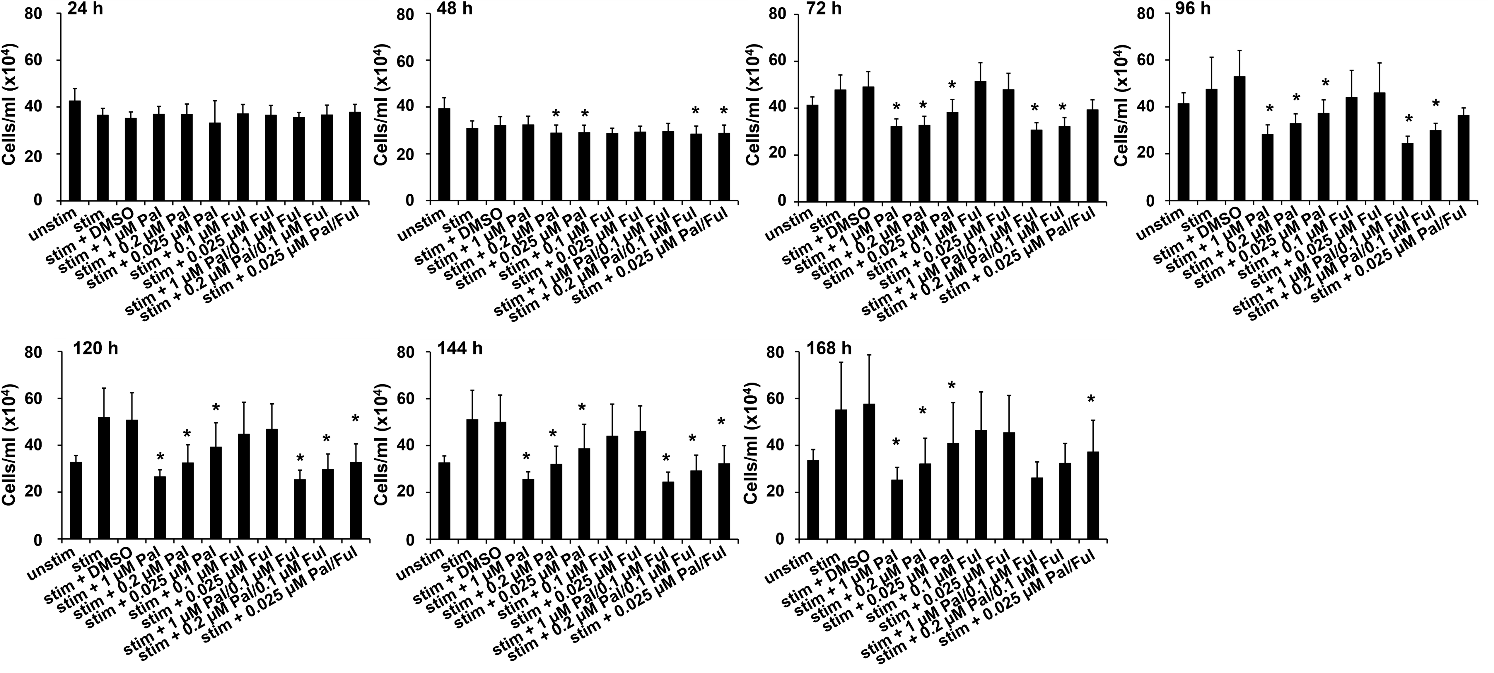
**

**Supplementary Figure 1.** Effect of palbociclib and fulvestrant on T cell expansion. eFluor^TM^ 670-stained T cells were stimulated by anti-CD3/CD28 beads and cultured in the presence or absence of palbociclib (daily addition), fulvestrant, or their combination at indicated concentrations for 24 h, 48 h, 72 h, 96 h, 120 h, 144 h, and 168 h. Cells were harvested and number of T cells was determined by flow cytometry. The results are depicted as the means ± SEM of four donors. (*p ≤ 0.05 compared to control sample “stim + DMSO”).


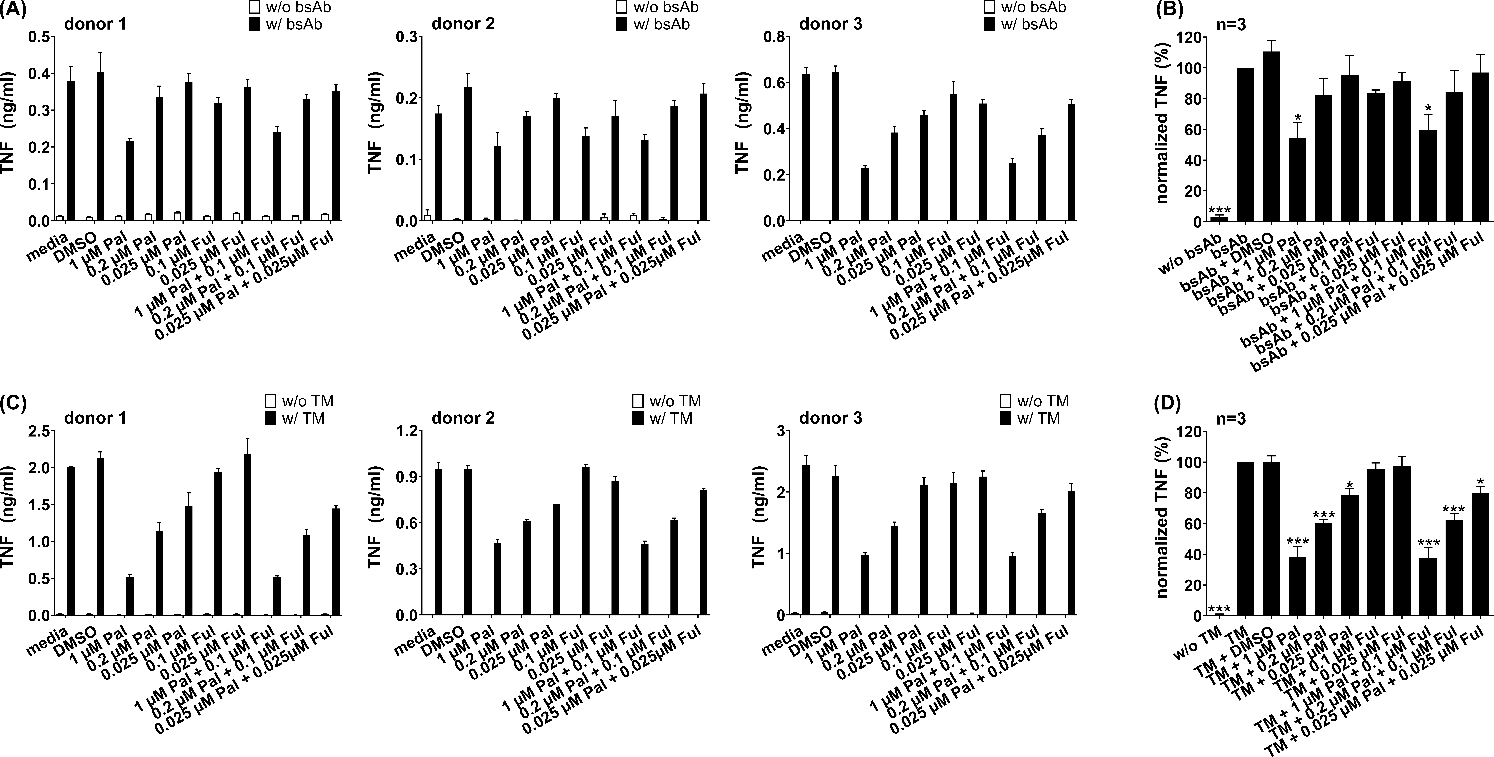


**Supplementary Figure 2.** Effect of palbociclib and fulvestrant on TNF release. PC3‑PSCA/PSMA Luc+ cells and **(A,B)** T cells or **(C,D)** UniCAR T cells were incubated with or without 30 nM of **(A,B)** CD3-PSCA bsAb or **(C,D)** PSCA TM in the presence or absence of palbociclib and/or fulvestrant. After 96 h, TNF concentrations in co-culture supernatants were analyzed by ELISA. **(A,C)** Each diagram shows average TNF concentration ± SEM of triplicates for one T cell donor. **(B,D)** Graphs summarize relative TNF release ± SEM of three different T cell donors. TNF concentrations in the presence of **(B)** T cells, tumor cells and bsAb (“bsAb”) or **(D)** UniCAR T cells, tumor cells and TM (“TM”) were equalized to 100%. (*p ≤ 0.05, ***p ≤ 0.001 compared to control sample “bsAb” or “TM”, respectively; One-way ANOVA with posthoc Dunnett multiple comparison test).


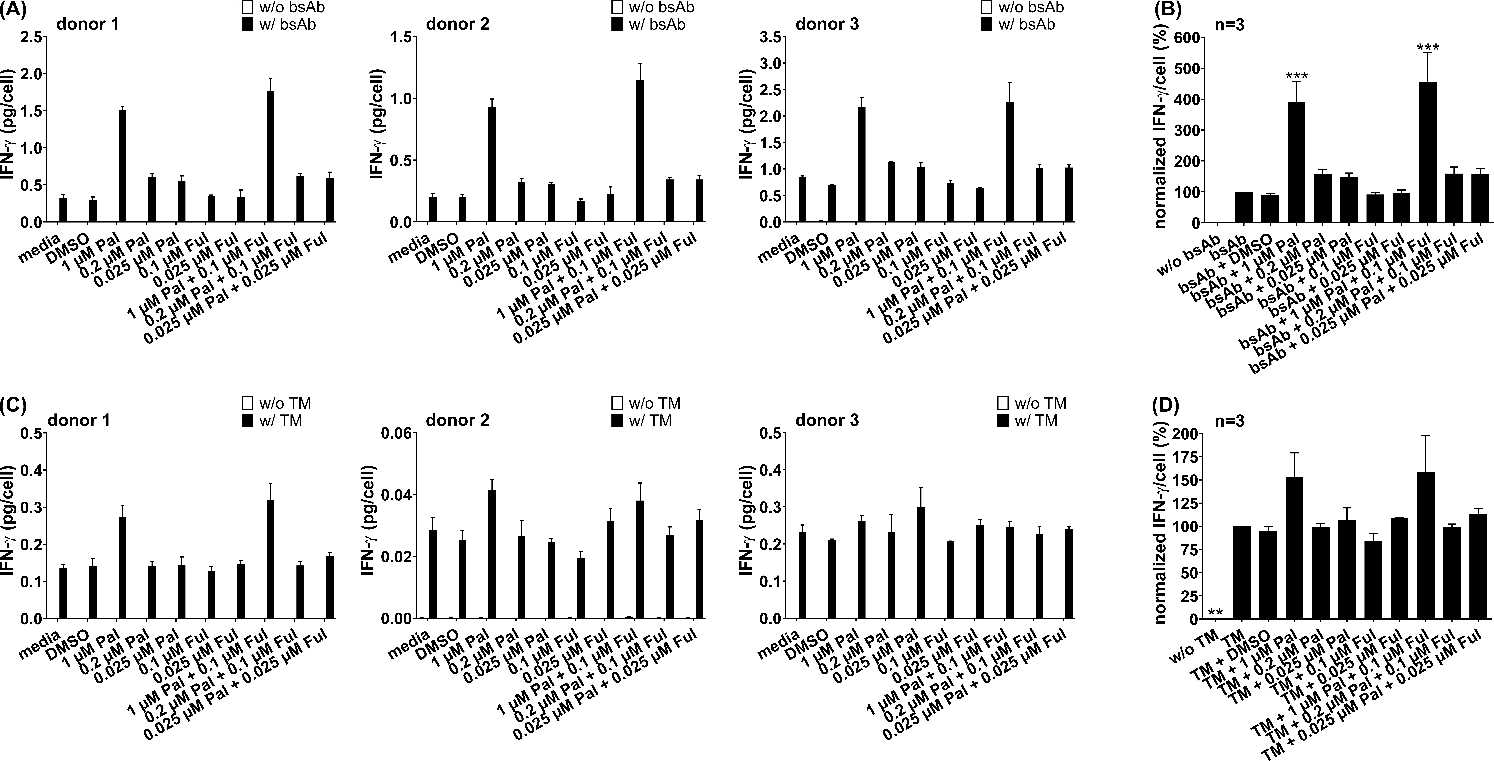


**Supplementary Figure 3.** Effect of palbociclib and fulvestrant on IFN-γ release per bsAb-engaged T cell or activated UniCAR T cell. PC3-PSCA/PSMA Luc+ cells and **(A,B)** eFluor^TM^ 670^+^ T cells or **(C,D)** eFluor^TM^ 670^+^ UniCAR T cells were incubated with or without 30 nM of **(A,B)** CD3-PSCA bsAb or **(C,D)** PSCA TM in the presence or absence of palbociclib and/or fulvestrant. After 96 h, IFN‑γ concentrations in co-culture supernatants and numbers of eFluor^TM^ 670^+^ T cells were determined. **(A,C)** Each diagram shows average IFN-γ concentration **(A)** per T cell or **(C)** UniCAR T cell ± SEM of triplicates for one T cell donor. **(B,D)** Graphs summarize relative IFN-γ release per cell ± SEM of three different T cell donors. IFN-γ concentrations per cell in the presence of **(B)** T cells, tumor cells and bsAb (“bsAb”) or **(D)** UniCAR T cells, tumor cells and TM (“TM”) were equalized to 100%. (**p ≤ 0.01, ***p ≤ 0.001 compared to control sample “bsAb” or “TM”, respectively; One-way ANOVA with posthoc Dunnett multiple comparison test).


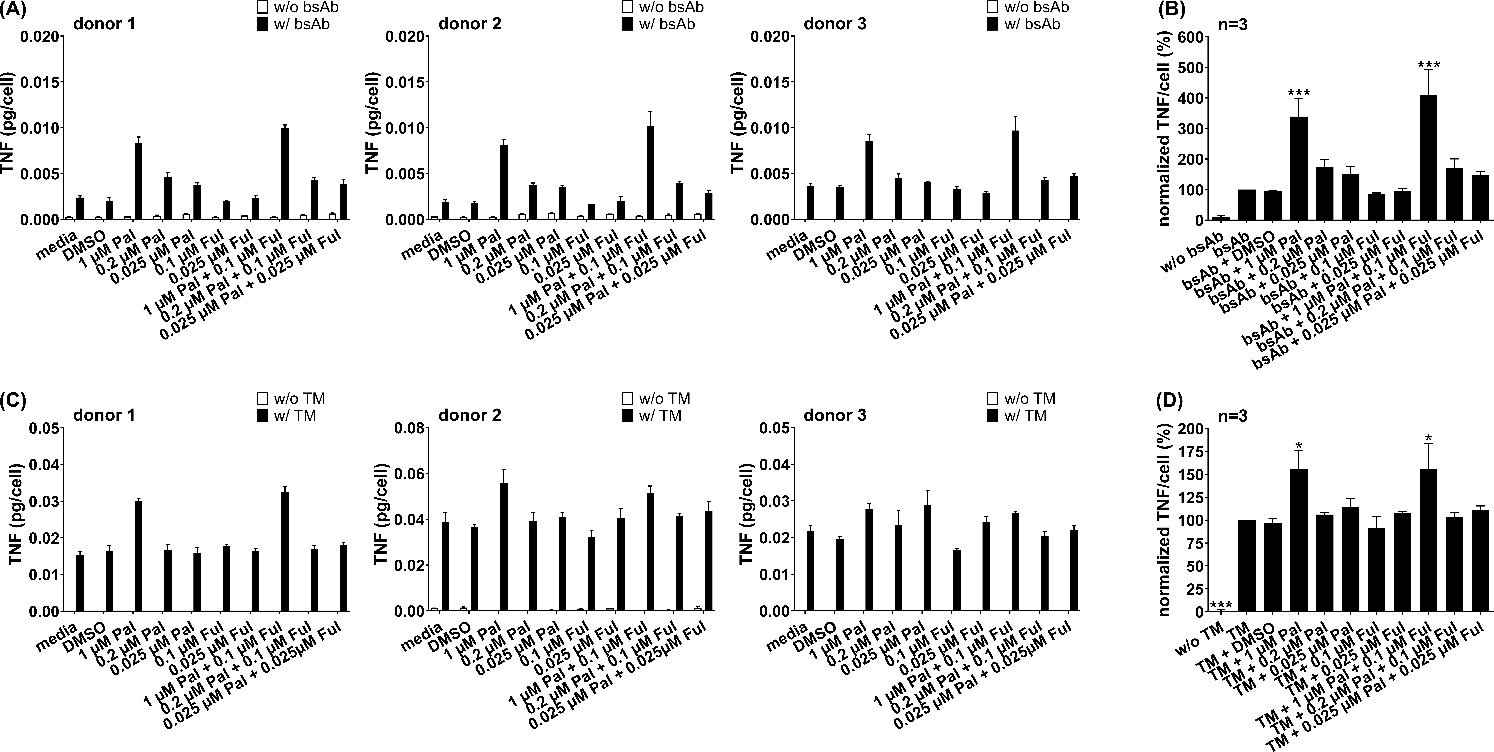


**Supplementary Figure 4.** Effect of palbociclib and fulvestrant on TNF release per bsAb-engaged T cell or activated UniCAR T cell. PC3-PSCA/PSMA Luc+ cells and **(A,B)** eFluor^TM^ 670^+^ T cells or **(C,D)** eFluor^TM^ 670^+^ UniCAR T cells were incubated with or without 30 nM of **(A,B)** CD3-PSCA bsAb or **(C,D)** PSCA TM in the presence or absence of palbociclib and/or fulvestrant. After 96 h, TNF concentrations in co-culture supernatants and numbers of eFluor^TM^ 670^+^ T cells were determined. **(A,C)** Each diagram shows average TNF concentration **(A)** per T cell or **(C)** UniCAR T cell ± SEM of triplicates for one T cell donor. **(B,D)** Graphs summarize relative TNF release per cell ± SEM of three different T cell donors. TNF concentrations per cell in the presence of **(B)** T cells, tumor cells and bsAb (“bsAb”) or **(D)** UniCAR T cells, tumor cells and TM (“TM”) were equalized to 100%. (*p ≤ 0.05, ***p ≤ 0.001 compared to control sample “bsAb” or “TM”, respectively; One-way ANOVA with posthoc Dunnett multiple comparison test).


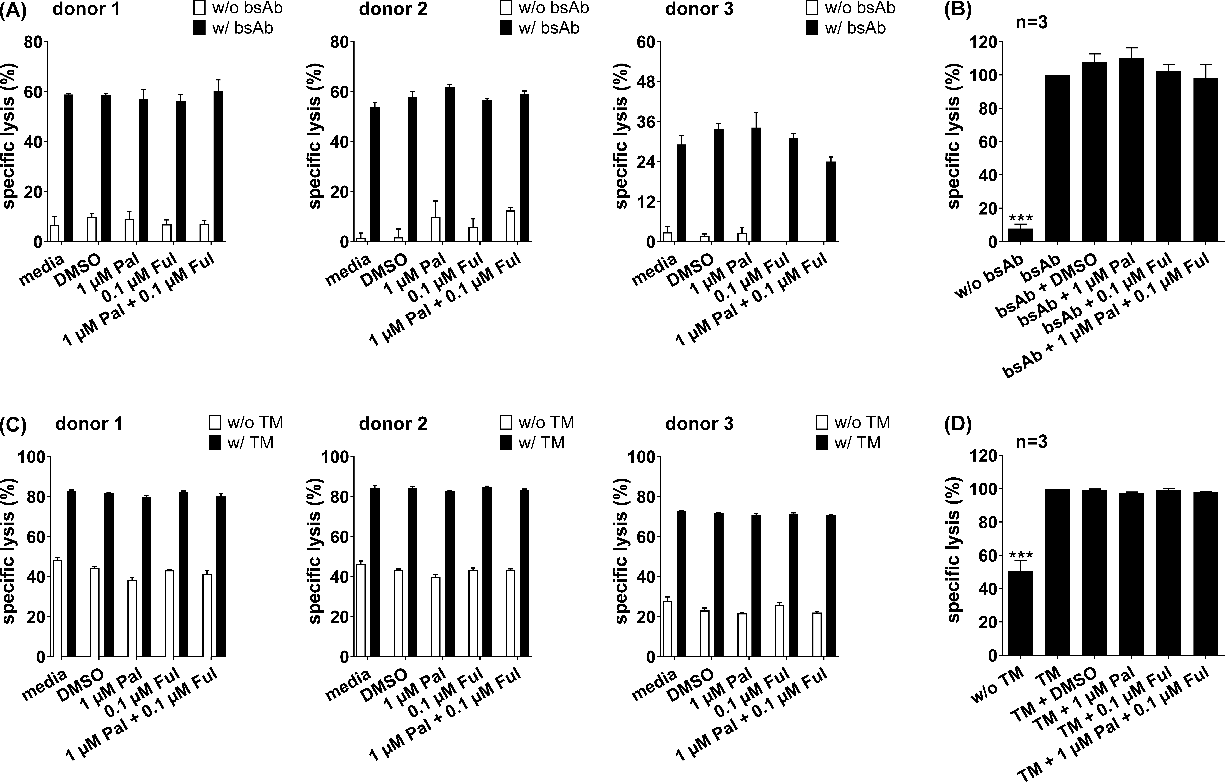


**Supplementary Figure 5.** Effect of palbociclib and fulvestrant on bsAb-mediated or UniCAR T cell-mediated tumor cell killing. PC3-PSCA/PSMA Luc+ cells and **(A,B)** T cells or **(C,D)** UniCAR T cells were incubated with or without 30 nM of **(A,B)** CD3-PSCA bsAb or **(C,D)** PSCA TM in the presence or absence of palbociclib and/or fulvestrant. After 8 h, tumor cell killing was calculated based on a luminescence-based killing assay. **(A,C)** Each diagram shows mean specific lysis ± SEM of triplicates for one T cell donor. **(B,D)** Graphs summarize relative tumor lysis ± SEM of three different T cell donors. Specific lysis in the presence of **(B)** T cells, tumor cells and bsAb (“bsAb”) or **(D)** UniCAR T cells, tumor cells and TM (“TM”) were equalized to 100%. (***p ≤ 0.001 compared to control sample “bsAb” or “TM”; One-way ANOVA with posthoc Dunnett multiple comparison test).
